# Addressing Electron Spins Embedded in Metallic Graphene Nanoribbons

**DOI:** 10.1021/acsnano.2c05673

**Published:** 2022-08-29

**Authors:** Niklas Friedrich, Rodrigo E. Menchón, Iago Pozo, Jeremy Hieulle, Alessio Vegliante, Jingcheng Li, Daniel Sánchez-Portal, Diego Peña, Aran Garcia-Lekue, José Ignacio Pascual

**Affiliations:** † CIC nanoGUNE-BRTA, 20018 Donostia-San Sebastián, Spain; ‡ Donostia International Physics Center (DIPC), 20018 Donostia-San Sebastián, Spain; ¶ CiQUS, Centro Singular de Investigación en Química Biolóxica e Materiais Moleculares, 15705 Santiago de Compostela, Spain; § Centro de Física de Materiales CSIC-UPV/EHU, 20018 Donostia-San Sebastián, Spain; ∥ Ikerbasque, Basque Foundation for Science, 48013 Bilbao, Spain

**Keywords:** graphene nanoribbons, magnetism, ballistic transport, on-surface synthesis, density functional theory, scanning tunneling microscopy

## Abstract

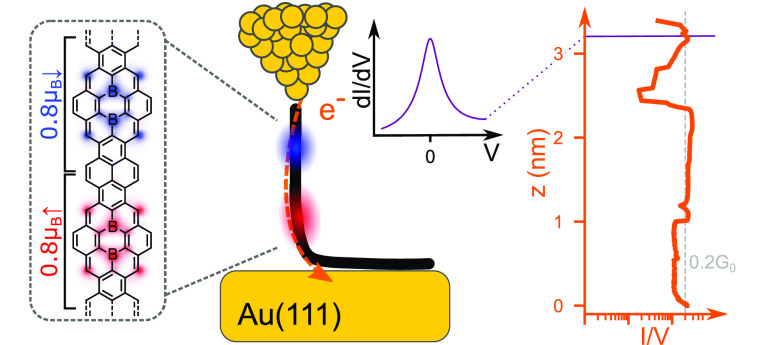

Spin-hosting graphene nanostructures are promising metal-free systems for elementary quantum spintronic devices. Conventionally, spins are protected from quenching by electronic band gaps, which also hinder electronic access to their quantum state. Here, we present a narrow graphene nanoribbon substitutionally doped with boron heteroatoms that combines a metallic character with the presence of localized spin 1/2 states in its interior. The ribbon was fabricated by on-surface synthesis on a Au(111) substrate. Transport measurements through ribbons suspended between the tip and the sample of a scanning tunneling microscope revealed their ballistic behavior, characteristic of metallic nanowires. Conductance spectra show fingerprints of localized spin states in the form of Kondo resonances and inelastic tunneling excitations. Density functional theory rationalizes the metallic character of the graphene nanoribbon due to the partial depopulation of the valence band induced by the boron atoms. The transferred charge builds localized magnetic moments around the boron atoms. The orthogonal symmetry of the spin-hosting state’s and the valence band’s wave functions protects them from mixing, maintaining the spin states localized. The combination of ballistic transport and spin localization into a single graphene nanoribbon offers the perspective of electronically addressing and controlling carbon spins in real device architectures.

Graphene nanoribbons (GNRs) are narrow stripes of graphene a few nanometer wide. In spite of graphene being inherently a semimetallic material, electronic correlations and confinement of their electrons into one dimension generally result in gapped band structures.^1,2^ Since the band gap depends on the GNR’s orientation, edge, and width,^2−6^ precise control of their semiconducting character can be achieved by on-surface synthesis methods.^6−10^

In the last years, it has been proposed that GNRs can also host localized spin states at specific positions of their carbon lattice, turning them into potential candidates for metal-free spintronic devices.^11−14^ Spin states have been found in GNRs and in graphene nanoflakes, mostly localized around zigzag edges and various types of defects.^15−35^ Two-terminal electronic transport measurements using a scanning tunneling microscope (STM) have demonstrated that spins can be addressed by electrons tunneling through the GNR’s band gap.^23,36^ Although a band gap favors spin localization, it restricts low-energy electron movement to distances of a few angstroms. This limits the integration of spin-hosting GNRs into spintronic devices. Ballistic transport through metallic GNRs^37−39^ would ease the implementation by facilitating the read out of the embedded spins.

Here, we report on the detection of localized spins in metallic GNRs realized by substitutionally doping a narrow band gap GNR with boron atoms in its interior. The boron heteroatoms turn the ribbon metallic and, at the same time, acquire a net magnetic moment. Density functional theory (DFT) calculations reveal that the spin is protected from the partially filled valence band (VB) by the different symmetry of the VB and the boron bands. Pentagonal defects, as those observed in the experiment, break the structural symmetry and open small hybridization gaps in the VB close to the Fermi level. We combined two-terminal transport experiments with differential conductance (d*I*/d*V*) spectroscopy to probe the electronic and magnetic properties of individual GNRs. Ballistic transport was stable over distances of several nanometers. The presence of a Kondo resonance proves access to the spin at a transport length of more than 3 nm.

## Results and Discussion

### Simulations of the Electronic Structure of a Metallic Graphene Nanoribbon

**Figure 1:**
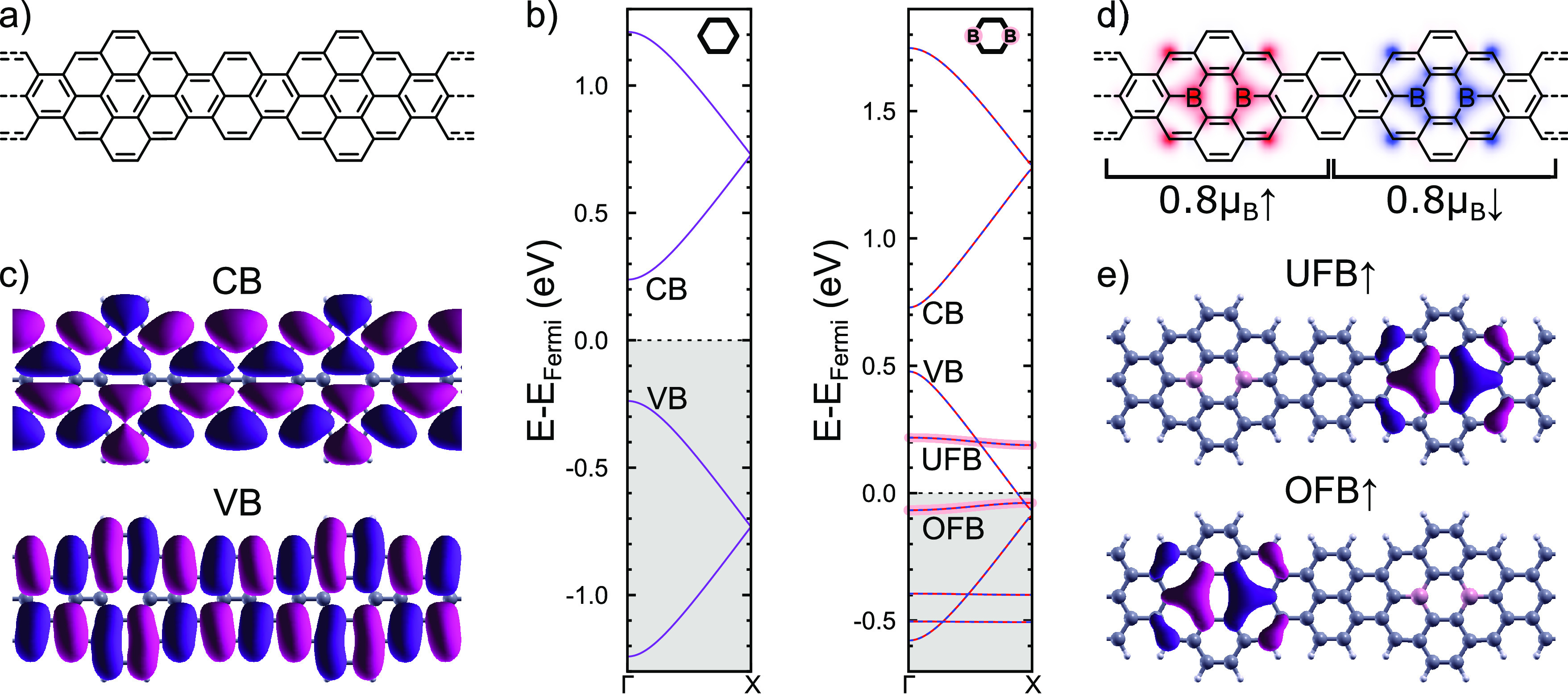
(a) Lewis structure of the proposed 575-aGNR without boron doping. (b) Spin-polarized DFT calculated band structure of the 575-aGNR and the 2B-575-aGNR using a doubled supercell like shown in panel (a). Boron character of the bands is indicated by a pink shadow. (c) DFT calculated wave functions at Γ of the CB and VB of the 575-aGNR. (d) Lewis structure of the 2B-575-aGNR shown on top of a color map representing the calculated spin polarization density. (e) DFT calculated wave functions at Γ of the spin-up unoccupied (UFB) and occupied (OFB) boron flat band of the 2B-575-aGNR.

The atomic structure of the investigated ribbon is derived from that of a 7-atom wide armchair GNR (7aGNR) with substitutional boron doping at periodic intervals.^35,40−46^ Here, we modified the edge structure and width of the undoped 7aGNR by periodically alternating five and seven carbon atom wide segments (575-aGNR, Figure 1a). DFT calculations of the electronic band structure, shown in Figure 1b, predict that the undoped GNR has a small band gap, with no spin polarization. Interestingly, the wave function of both valence and conduction bands is antisymmetric with respect to the central axis of the ribbon (Figure 1c), in contrast with the symmetric character of frontier bands in the related 7aGNR.^44^ This change in the bands’ symmetry turns out to be crucial to understand the effect of boron substitution inside the GNR.

Substituting the two central carbon atoms in the wider segments with boron atoms (as shown in Figure 1d) creates two boron-rich flat bands. These bands originate from pure boron orbitals and have no topological character, unlike the flat bands of 2B-7aGNRs.^23,44^ The band structure of the 2B-575-aGNR, also shown in Figure 1b, reveals a significant charge transfer from its VB to the boron bands, resulting in an occupied flat band (OFB), hosting approximately two electrons, and an unoccupied flat band (UFB). The VB is partially depopulated and becomes metallic.

The boron flat bands and VB cross without opening a gap, as shown in Figure 1b, revealing a negligible mixing. This is a consequence of the different symmetries of their wave functions (Figure 1c,e, Supporting Information (SI) Figure 14). The boron flat bands, localized around the boron atoms, are symmetric with respect to the central ribbon axis, while the VB is antisymmetric. The orthogonality between the VB and boron flat bands allows electrons in the VB to propagate unperturbed along the ribbon,^42^ resulting in a metallic band and maintaining the boron states localized around the diboron impurities. DFT finds a magnetic moment of 0.8μ_B_, close to spin *S* = 1/2, associated with the OFB that is localized around each 2B-unit. Spin moments in adjacent 2B-units tend to antialign, as shown in Figure 1d, so the periodic system shows no net spin polarization. For further details on the boron character of the flat bands and the influence of the ribbon width on the magnetic moment see SI Figures 11 and 13.

### On-Surface Synthesis of 2B-575-aGNRs

**Figure 2:**
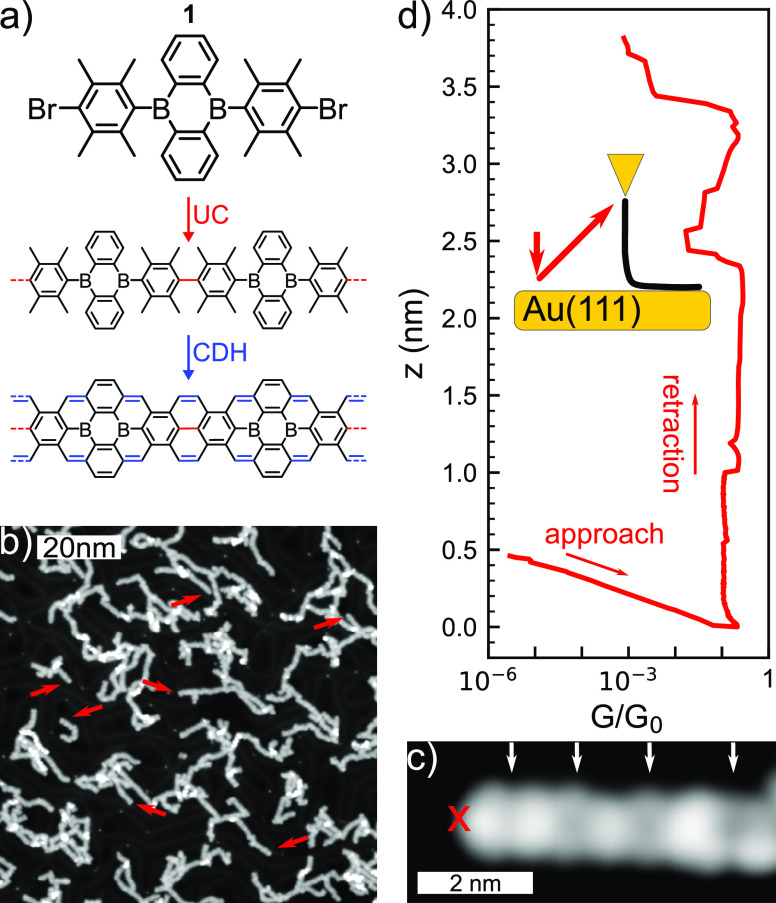
(a) Molecular precursor **1** and the targeted two step reaction for synthesizing 2B-575-aGNRs via Ullmann coupling and cyclodehydrogenation. (b) STM topography image (*V* = 1 V, *I* = 30 pA). Straight 2B-575-aGNRs are indicated by red arrows. (c) STM topography image (*V* = −300 mV, *I* = 30 pA) of a four precursor unit long 2B-575-aGNR. The positions of boron doping are indicated by white arrows. The red cross indicates the position from where the GNR is lifted for the transport experiment. (d) *G*(*z*, *V* = 10 mV) for the GNR presented in (c). The conductance is independent of *z* up to *z* ≈ 2.2 nm. The inset is a schematic drawing of the experimental setup.

Based on the intriguing properties predicted by DFT calculations, we decided to explore the on-surface synthesis and characterization of this boron-doped GNR. A retrosynthetic analysis identified the compound shown in Figure 2a as the ideal molecular precursor, which might lead to the formation of 2B-575-aGNRs by sequential Ullmann coupling and cyclodehydrogenation reactions on a Au(111) substrate. The molecular precursor was obtained by solution chemistry in one step from easily available starting materials (see SI “Synthesis of the molecular precursor” section for details) and sublimated *in situ* on Au(111). Polymerization occurs at 250 °C, a higher temperature than those for other systems,^7,41^ and close to the onset of cyclodehydrogenation of the polymer. The presence of the precursor’s bulky methyl groups increases the energy barrier for the formation of metal organic complexes,^47^ which have been shown to facilitate the on-surface Ullmann coupling.^48^ As a consequence, the Ullmann coupling requires a higher temperature for activation. We annealed the sample to 300 °C to achieve a high amount of planar ribbons. The resulting structures were mostly curved and interlinked ribbons, as seen in Figure 2b, with a few short and straight segments (red arrows). Figure 2c shows an STM image of a single straight 2B-575-aGNR segment, where four boron doping sites can be identified as wider and darker segments of the ribbon.

### Two-Terminal Electronic Transport Measurements

We studied the electronic transport through a GNR suspended between the tip and sample of an STM.^18,23,36,49−51^ To reach this two-terminal configuration, we positioned the tip above the apex of the 2B-575-aGNR (red cross in Figure 2c) and approached the tip toward the substrate until a sudden increase in the current indicated the formation of a bond between tip and ribbon. Then, we retracted the tip following a leaned trajectory along the backbone of the ribbon (see Experimental Section for details on the procedure). This procedure lifts the 2B-575-aGNR partially from the Au(111) and electronically decouples the free-standing segment from the metal.

Electronic transport measurements through the lifted ribbons confirm that they behave as ballistic conductors. As shown in Figure 2d, the linear conductance *G*(*z*) of the ribbon remains constant for several nanometers while the tip is retracted. The constant conductance contrasts with the exponentially decaying conductance found for semiconducting ribbons.^23,36,49,52^ In a ballistic conductor, the electron transmission  remains constant as a function of its length, and the conductance per channel amounts to , where *G*_0_ = *e*^2^/*πℏ* = 77.5 μS is the conductance quantum. In the results shown in Figure 2, we observe high conductance values around  remaining constant for more than 2 nm of GNR elevation. The electron transmission smaller than *G*_0_ is probably caused by the finite contact resistance between tip and GNR.^53,54^ At some points we find small variations of the conductance around 0.1*G*, which are consistent with atomic-scale rearrangements of the GNR-electrode contacts when additional borylated units detach from the surface.^49,55^ The ballistic electron transport found here reflects the existence of scattering free transmission channels in free-standing 2B-575-aGNRs, in agreement with the boron induced metallic character revealed by our DFT calculations in Figure 1.

### Observation of the Kondo Effect in Ballistic Ribbons

**Figure 3:**
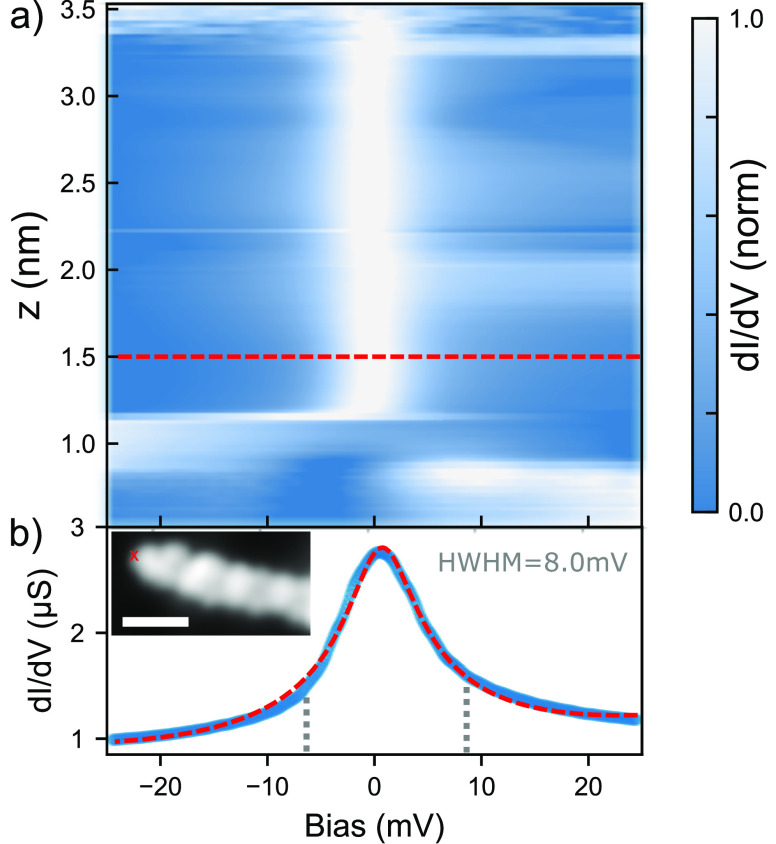
(a) Normalized d*I*/d*V*(*V*, *z*) map (see Experimental Section for details on normalization procedure). A zero-bias resonance appears in the spectra for *z* > 1.2 nm. (b) One example d*I*/d*V* spectrum (blue) fitted with a Frota function^56^ (red dashed line). The spectrum was taken at *z* = 1.5 nm. Inset: STM topography image (*V* = −300 mV, *I* = 30 pA, scale bar is 2 nm). The red cross indicates the position from where the GNR is lifted.

Figure 3a shows a d*I*/d*V*(*V*, *z*) spectral map obtained by measuring d*I*/d*V* spectra during the lift of a 2B-575-aGNR (inset Figure 3b). A narrow zero-bias resonance (HWHM = 8.0 mV) appears suddenly at *z* = 1.2 nm and prevails up to *z* ≥ 3.5 nm during the lifting procedure (see SI Figure 3 for simultaneously recorded *G*(*z*)). We interpret this resonance as a manifestation of the Kondo effect in the electronic transport through the ribbon.^57^ The Kondo resonance is the fingerprint of a spin state weakly coupled to an electron bath.^15,18,21,23,58−60^ Here, it is observed for more than 2 nm during the GNR elevation, hinting that the Kondo screening is not simply mediated by electrons at the surface.^23^ We suggest that the metallic band of the ribbon is responsible for the screening of the localized magnetic moments.

In the transport experiments presented in Figures 2 and 3, we found electronic and magnetic fingerprints that are consistent with our DFT calculations of the 2B-575-aGNR. All 19 ribbons explored in this manner show similar results, in every case reproducing segments of constant conductance associated with ballistic transport, and Kondo resonances. However, many ribbons also showed a stepwise decrease in their conductance plots at some elongations, which we attribute to the presence of defects in their structure.

### Role of Atomic Defects on the Nanoribbons

**Figure 4:**
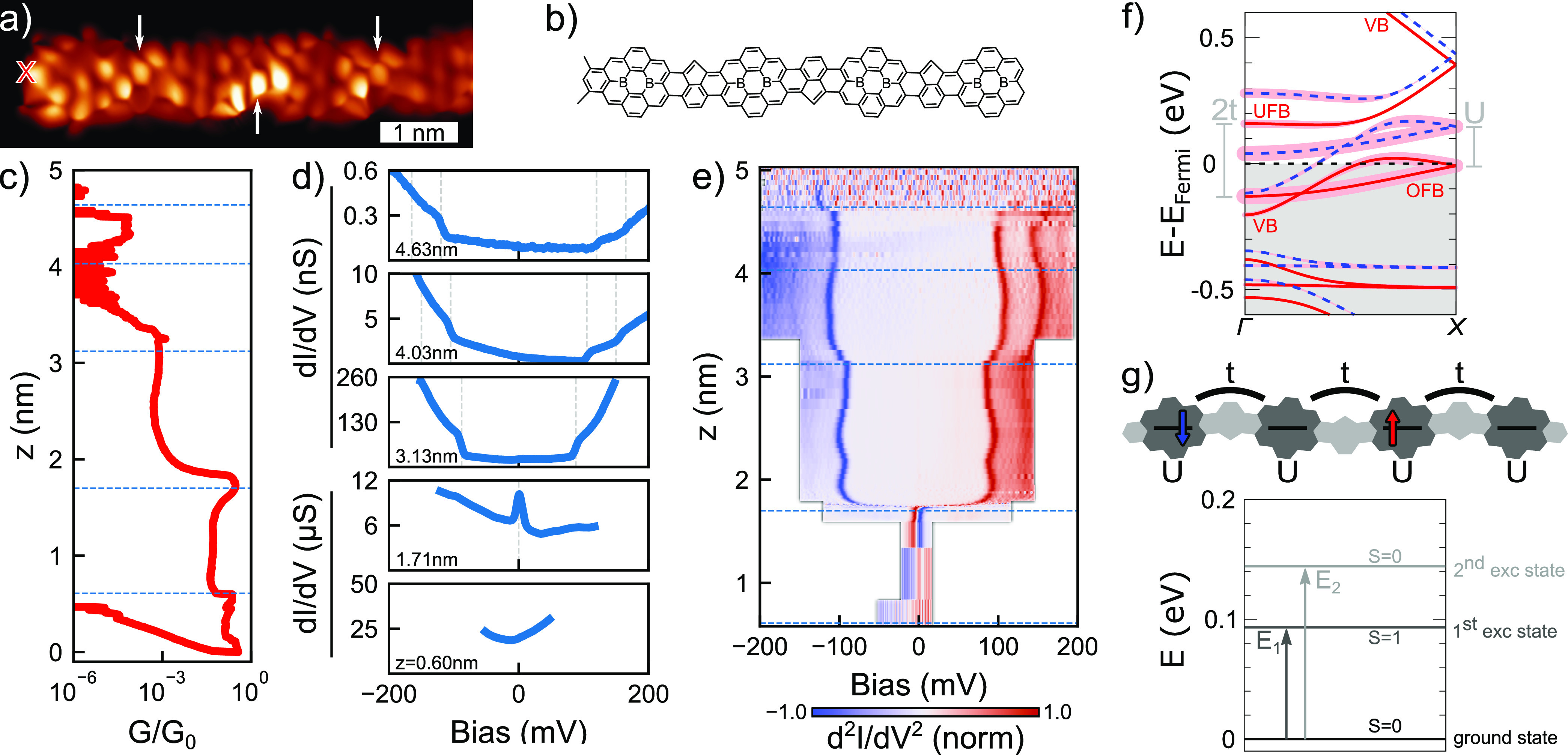
(a) Bond-resolved constant height current image (*V* = 5 mV). The 2B-units alter the contrast due to buckling of the ribbon. Pentagons are indicated by white arrows. The red cross indicates the position from where the ribbon is lifted. (b) Lewis structure of the 2B-575*-aGNR in (a). (c) Linear conductance *G*(*z*, *V* = 10 mV) obtained while lifting the ribbon presented in (a). Some ballistic behavior is retained. (d) d*I*/d*V* spectra at selected *z*. The values are indicated and correspond to the blue, dotted lines in (c) and (e). A single Kondo-resonance at *z* = 1.71 nm indicates the presence of a spin *S* = 1/2. For larger *z* inelastic excitations dominates the spectra. (e) Normalized d^2^*I*/d*V*^2^(*V*, *z*) map obtained by numerical differentiation (see Experimental Section for details on normalization procedure). (f) DFT calculated band structure of periodic 2B-575*-aGNR. Notice that in this case the unit cell necessarily contains two 2B-units. Red and blue bands correspond to spin up and down, respectively. Boron character of the bands is indicated by a pink shadow. The corresponding band from the 2B-575-aGNR is indicated for the spin up band. (g) Hubbard model used to calculate spin-excitations of the finite sized system and its energy spectrum for two electrons that are delocalized across four hopping sites. *U* = 155.9 meV, *t* = 133.9 meV, obtained from (f).

To detect the presence of atomic-scale defects in 2B-575-aGNRs, we measured constant height current images using a CO-functionalized tip.^61,62^ The image of a nanoribbon composed of 4 molecular units (*i.e.*, 4 boron dimers) is shown in Figure 4a. It is consistent with a ribbon containing a sequence of pentagonal rings in its carbon backbone at the position of Ullmann coupling (white arrows in Figure 4a). The extracted Lewis structure of the ribbon (referred to as 2B-575*-aGNR) is shown in Figure 4b. Pentagonal rings are known to appear when methyl groups of the precursor detach during the polymerization reaction.^17,24,63^ This type of defect at the linking position is the most common structure we find in an analysis of 16 ribbons (see also SI Figure 4).

To unravel the effect of the atomic defects on the electronic transport of 2B-575-aGNRs, we performed two-terminal transport measurements on the ribbon shown in Figure 4 in a suspended geometry. As depicted in Figure 4c, the conductance *G*(*z*) decreases stepwise with increasing *z*. The conductance steps are spaced by Δ*z* ∼ 1.4 nm, matching with the distance between two diboron sites. This suggests that they appear when a new precursor unit is inserted in the free-standing part of the ribbon.^55^

Between conductance steps, constant conductance plateaus unveil that some ballistic behavior is retained. However, now, we found three qualitatively different regimes, depicted in Figure 4d. First, we observed a metallic-like behavior, with a flat d*I*/d*V* ∼ 0.3*G*_0_ signal persisting until *z* = 0.6 nm, where the first conductance steps appears. Upon further tip retraction, a zero-bias resonance similar to the one shown in Figure 3 appears in the spectra. Again, this indicates that a localized spin appears in the free-standing segment of the ribbon. This Kondo feature disappears at *z* = 1.75 nm, coinciding with a second step in the linear conductance plot of Figure 4c. Above this *z* value, spectra exhibit two bias-symmetric d*I*/d*V* steps, characteristic of inelastic electron tunneling (IET) excitations.

To follow the IET spectral evolution during the retraction, we show in Figure 4e a normalized d^2^*I*/d*V*^2^(*V*, *z*) map. We observe that at *z* = 1.75 nm the Kondo resonance splits gradually in ≤1 Å and converts into IET steps (see SI Figure 5). Above this value, the steps are observed for more than 3 nm retraction with small variations of their excitation energy. A fainter d*I*/d*V*-step, at approximately 45 mV larger bias voltage, can also be observed in the spectra above 3 nm. The continuous evolution from Kondo to IET excitations suggests that a complex spin texture exists in the 2B-575*-aGNR.

DFT calculations for periodic 2B-575*-aGNRs revealed that the presence of the pentagonal rings in the ribbon has two important implications. First, they break the structural symmetry of the GNR, mixing the wave functions of boron flat bands and VB. Now, the band structure in Figure 4f (SI Figure 12) shows avoided crossings of both UFB and OFB with the VB, characteristic of a small hybridization. Second, the removal of a carbon atom from the ribbon effectively injects another hole and lowers the occupation of both the VB and the OFB. Since the VB still crosses the Fermi level, the ribbon preserves its metallic character. DFT pictures the depopulation of the OFB as a mean delocalization of an electron over several 2B-units and a smaller net magnetic moment associated with each diboron unit (∼0.5μ_B_/2B).

To interpret the experimental IET signal, we explored different magnetic states obtained by DFT simulations of finite ribbons, like the one presented in Figure 4g (SI Figures 8–10). The DFT results indicate that the system cannot be simply treated as Heisenberg-like Hamiltonian due to the electron delocalization. In fact, DFT significantly underestimates the excitation energies as compared to the measured IET spectra and does not fully capture the relevant physical mechanisms behind the inelastic steps.

Given that the hybridization between the VB and the boron flat bands is small (notice in Figure 4f the 1 to 10 ratio between the size of the hybridization gaps and the VB bandwidth), a valid approximation is treating VB and OFB as two different subsystems, disregarding excitations that imply charge-transfer between them. The observed spectral steps can then be attributed to inelastic excitations in the OFB/UFB subsystem induced by conduction electrons propagating through the VB. To describe the excitation spectrum of the boron flat bands, we use a Hubbard model with parameters *t* and *U* obtained from the DFT band structure in Figure 4f. This simple model can be exactly solved and approximately accounts for electron correlations in the excitation spectrum. Based on the OFB’s occupation observed in DFT calculations (see Figure 4f), we consider the probable case of two electrons distributed over four electron sites.

Exact diagonalization of the Hubbard Hamiltonian leads to the energy spectrum presented in Figure 4g. The ground state is a singlet combination of the two spins. The first excited state is a triplet state located at *E*_1_ = 94 meV, which matches reasonably well with the energy of the first excitation step in our experiments. Furthermore, the model also finds an excited singlet state at *E*_2_ = 145 meV, in strong coincidence with the second spectral IET step. The observed agreement between the calculated and experimentally observed excitation energies indicates the IET signals can be due to electron–hole pair excitations of a partially populated flat band, a similar excitation process to the one recently observed on small molecules on insulating layers.^64^ This interpretation is supported by the fact that the excitation energies in the model are dominated by the hopping parameter between two 2B-sites *t* and are stable against changes of *U* (SI Figure 6) or electron occupation (SI Figure 7). This explains why the step values are relatively independent from the length of the free-standing segment. The model unveils a probable source of scattering phenomena in the electronic transport, which, also, can be responsible for the stepwise decrease of the linear conductance found when a new diboron unit is lifted from the surface (Figure 4c).

## Conclusions

We have presented a graphene nanoribbon that combines a metallic transport band with localized spins inside and described experimental fingerprints of both the metallic character and the spin-polarized states. The ribbon is fabricated by substitutionally doping the narrow-band gap 575-aGNR with diboron units. DFT simulations unveiled that the dispersive VB becomes partially depopulated by donating electrons to flat bands formed by the diboron units. As a result, single spins emerge localized at every diboron unit, protected from the VB by their different wave function symmetry. Two-terminal transport experiments through free-standing GNRs placed between tip and sample of an STM demonstrated ballistic electron transport, transmitting electrons with a constant value of 0.2*G*_0_ for a free-standing segment length of a few nanometers. Simultaneously, the differential conductance spectroscopy in the transport configuration unraveled the spin localization by revealing a Kondo resonance that was stable during the elevation of the GNR from the surface.

In addition to the ideal case, we found that atomic defects such as pentagonal rings in the structure, frequently found in our experiments, enable a small wave function mixing between VB and the boron flat bands and partially depopulate both bands. The effect of these pentagonal defects in the transport is drastic, because it enables a finite interaction between adjacent diboron units and delocalizes the electrons along the partially occupied flat band. We found that the ballistic character of the ribbon partly survives in the presence of pentagonal defects, but a new inelastic excitations appears in the spectra, accompanying a stepwise decrease of the linear conductance with ribbon elevation. Through a simple Hubbard model, parametrized with results from the DFT simulations, we found that the inelastic spectral features can be attributed to excitation of the many-body states of the partially depopulated flat band. These results thus demonstrate that the 2B-575-aGNR represents an ideal molecular system to import flat band phenomena into one-dimensional graphene nanoribbons, envisioning the study of the underlying electron transport phenomena present in these correlated systems.

## Experimental Section

### Sample Preparation

A Au(111) single crystal was prepared by Ne^+^ sputtering and successive annealing at *T* = 450 °C under ultrahigh vacuum conditions. The precursor molecules were sublimated *in situ* from a Knudsen cell at a temperature of 220 °C. Afterward, the gold was annealed to *T* = 180 °C for 10 min and flashed to *T* = 300 °C for 1 min. The samples were analyzed in a custom-made low-temperature STM at 5 K. The figures presenting experimental data were prepared using WSxM^65^ and the python matplotlib libary^66^ using perceptual continuous color scales.^67^

### Details on the Lifting Procedure

For lifting the nanoribbons, the tip was stabilized in close proximity to the apex above Au(111) at *V* = 1 V, *I* = 30 pA. Next, the feedback controller was switched off, the bias reduced to *V* = 10 mV and the current gain set to 10^6^. Only afterward the tip is placed above the termination of the GNR and approached slowly toward the sample monitoring the current. We used a custom built interface to control the tip movement in *x*, *y*, and *z* and simultaneously record the tip movement, the current, the bias, and the lock-in signal during the whole retraction process. A step-like increase in the current, typically after approaching ∼0.4 nm, indicates the formation of the tip-apex bond. From there, the tip is retracted in a trajectory following initially an up to 45° angle with respect to the surface normal along the ribbon backbone. The angle is reduced continuously with retraction, reaching 0° for larger *z* values, *i.e.*, pulling perpendicular to the surface. The trajectory was slightly adapted for each ribbon. We found no influence of the lateral displacement of the tip on d*I*/d*V* spectroscopy.

### Differential Conductance Measurements

Spectroscopic d*I*/d*V* measurements were performed using an external lock-in amplifier with frequency *f* = 867.6 Hz, time constant τ = 30 ms, and modulation *V*_mod_ = 2 mV. To record the differential conductance maps presented in Figures 3 and 4, we stabilized the tip at a desired *z* and performed one d*I*/d*V* spectroscopy. Afterward, we moved the tip by Δ*z* = 25 pm (Figure 3) and Δ*z* = 10 pm (Figure 4) and repeated the procedure. The *G*(*V*, *z*) map in Figure 3a is normalized for each *z* = *z*_0_ separately, using the formula *G*_norm_(*V*, *z*_0_) = (*G*(*V*, *z*_0_) – *G*_min_)/(*G*_max_ – *G*_min_), where *G*(*V*, *z*_0_) is the differential conductance and *G*_max(min)_ the maximum (minimum) value of *G*(*V*, *z*_0_). The d^2^*I*/d*V*^2^ map in Figure 4e is normalized equivalently using , where *G*′ is the derivative of the conductance and  is the minimum (maximum) of *G*′.  was used for negative (positive) values of *G*′(*V*).

### Density Functional Theory Calculations

First-principles electronic structure calculations were performed using DFT as implemented in the SIESTA software package.^68,69^ The van der Waals density functional by Dion *et al.*(70) with the modified exchange correlation by Klimeš, Bowler and Michaelides^71^ was used. The valence electrons were described by a double-ζ plus polarization (DZP) basis set with the orbital radii defined using a 54 meV energy shift,^69^ while the core electrons were described using norm-conserving Trouillers–Martins pseudopotentials.^72^ For integrations in real space,^69^ an energy cutoff of 300 Ry was used. The smearing of the electronic occupations was defined by an electronic temperature of 300 K with a Fermi–Dirac distribution. The self-consistency cycles were stopped when variations on the elements of both the density matrix and the Hamiltonian matrix were smaller than 10^–4^ eV. In order to avoid interactions with periodic images from neighboring cells, systems were calculated within a simulation cell where at least 50 Å of vacuum space was considered. Variable cell relaxations and geometry optimizations were performed using the conjugate gradient method using a force tolerance equal to 10 meV Å^–1^ and 0.2 GPa as stress tolerance. For periodic ribbons, a 40 k-point mesh along the GNRs’ periodic direction was used.

## Supporting Information Available

Synthesis of molecular precursors; complementary experimental data referenced in the main paper; four sites Fermi-Hubbard model; complementary DFT calculations (PDF)
