# Supplementary material for: Addressing Electron Spins Embedded in Metallic Graphene Nanoribbons
Source: ACS Nano. 2022 Aug 29;16(9):14819–26. doi: 10.1021/acsnano.2c05673 (PMC9527809; doi:10.1021/acsnano.2c05673)
Supplement: Supplementary file 1 — nn2c05673_si_001.pdf [file nn2c05673_si_001.pdf]

Addressing electron spins embedded in metallic graphene nanoribbons - Supporting Information

Niklas Friedrich,[*,†] Rodrigo E. Menchón,[‡] Iago Pozo,[¶] Jeremy Hieulle,[†] Alessio Vegliante,[†] Jingcheng Li,[†] Daniel Sánchez-Portal,[‡,§] Diego Peña,[*,¶] Aran Garcia-Lekue,[*,‡,∥] and José Ignacio Pascual[*,†,∥]

†*CIC nanoGUNE-BRTA, 20018 Donostia-San Sebastián, Spain*

‡*Donostia International Physics Center (DIPC), 20018 Donostia-San Sebastián, Spain*

¶*CiQUS, Centro Singular de Investigación en Química Biolóxica e Materiais Moleculares, 15705 Santiago de Compostela, Spain*

§*Centro de Física de Materiales CSIC-UPV/EHU, 20018 Donostia-San Sebastián, Spain*

∥*Ikerbasque, Basque Foundation for Science, 48013 Bilbao, Spain*

* E-mail: n.friedrich@nanogune.eu; diego.pena@usc.es; wmbgalea@ehu.eus; ji.pascual@nanogune.eu

Synthesis of the molecular precursor

General methods

Reactions were carried out under argon using oven-dried glassware. Et_2O and toluene were purified in a MBraun SPS-800 Solvent Purification System. 9,10-dibromo-9,10-diboraanthracene (DBA) was prepared following a published procedure[1] and stored in a glovebox. Other commercial reagents were purchased from ABCR GmbH or Sigma-Aldrich and were used without further purification. TLC was performed on Merck silica gel 60 F254 and chromatograms were visualized with UV light (254 and 365 nm). Column chromatography was performed on Merck silica gel 60 (ASTM 230-400 mesh). ^{1}H NMR spectra were recorded at 300 MHz (Varian Mercury-300 instrument).

Synthesis of precursor molecule

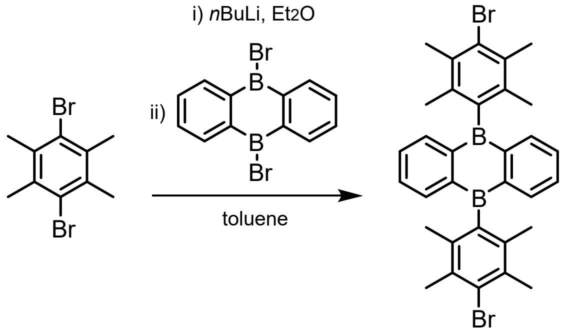

Figure 1: Synthetic route to obtain the boron-doped GNR precursor.

To a solution of 9,10-dibromodurene (174 mg, 0.60 mmol) in dry Et_2O (0.04 M) at −78 °C in a Schlenk tube, n-BuLi (2.5 M in hexane, 0.63 mmol) was dropwise added. Subsequently, the reaction mixture was stirred at 0 °C for 20 min and cooled again to −78 °C. Then, another solution of 9,10-dibromo-9,10-diboraanthracene (DBA, 100 mg, 0.30 mmol) in dry toluene (0.02 M) was dropwise added at −78 °C and allowed to warm up to rt°C for 18. Then, the solvent was removed under reduced pressure and the crude was purified by column chromatography (SiO_2, $CHCl_3$:hexane 1:1, Rf = 0.9). The solid obtained was washed with hexane and

centrifuged affording 5,10-bis(4-bromo-2,3,5,6-tetramethylphenyl)-5,10-dihydroboranthrene as a white solid (27 mg, m.p.: > 350 decomp., 15% yield). ^{1}H NMR (300 MHz, $CDCl_3$): 7.59 (m, 4H), 7.47 (m, 4H), 2.44 (s, 12H), 2.03 (s, 12H) ppm. MS (APCI), m/z: 598 (M+, 100). HRMS (APCI), m/z found: 596.1064 (calc. for $C_{32}H_{32}B_2Br_2$: 596.1051).

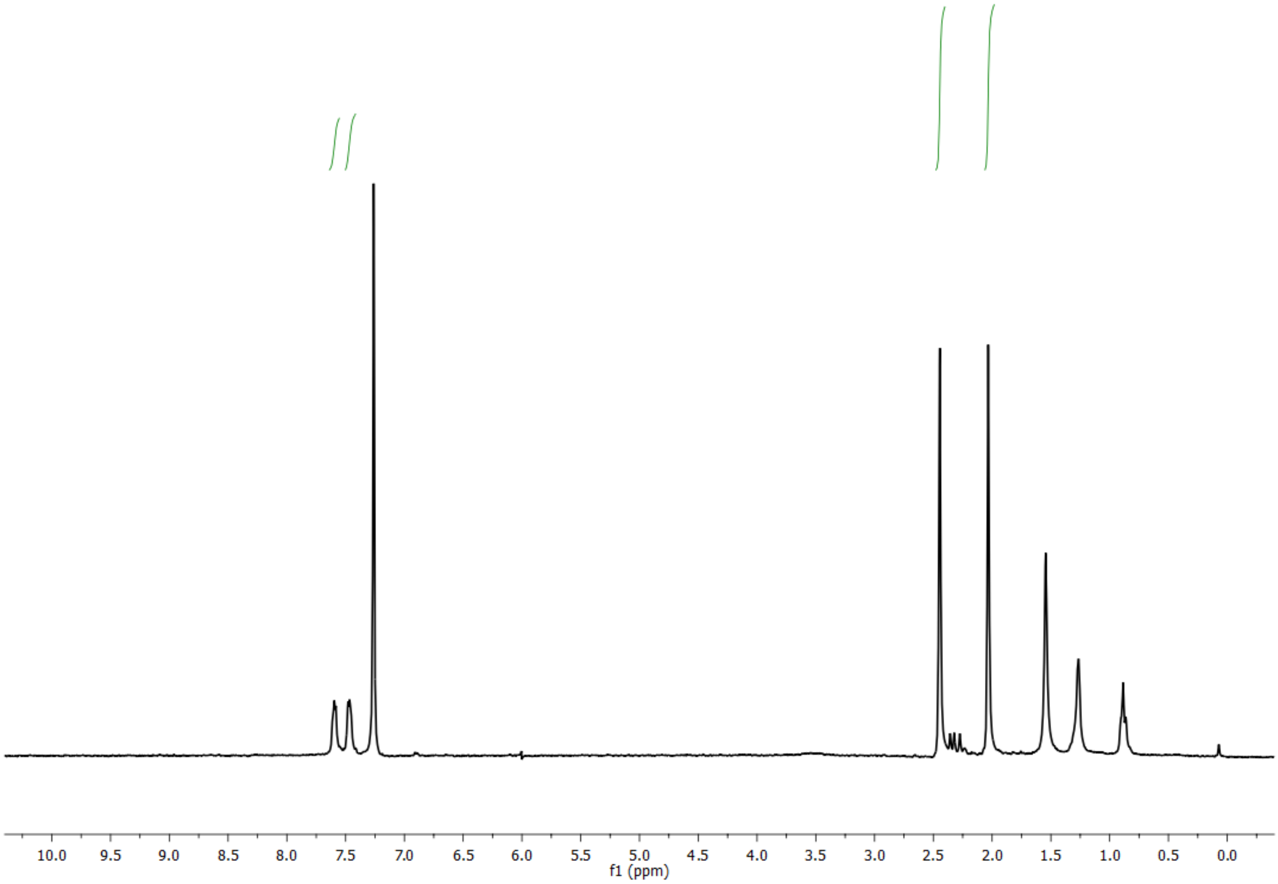

Figure 2: ^{1}H NMR of the boron-doped GNR precursor in $CDCl_3$ at room temperature

Complementary experimental data

Conductance - Distance Plot

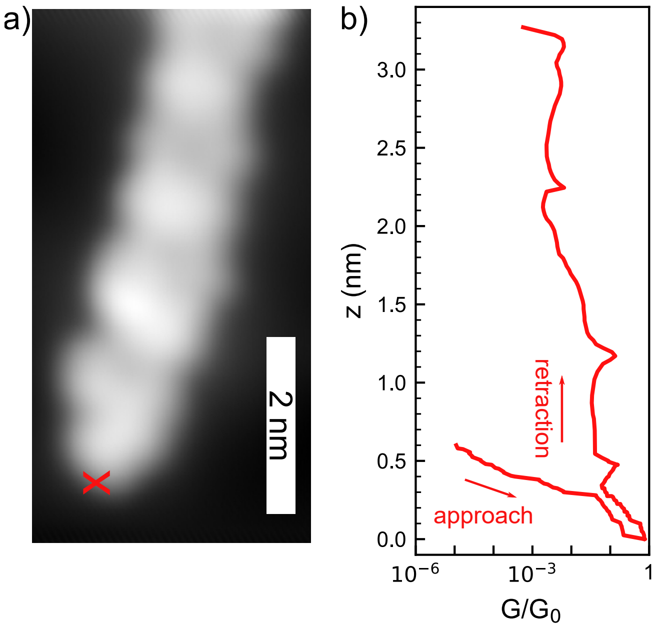

Figure 3: (a) STM topography image ($V = -300\,\text{mV}$, $I = 30\,\text{pA}$) of a 2B-575-aGNR. It is the same image as Figure 3b of the main text and only shown for reference. (b) $G(z, V = 10\,\text{mV})$ for the GNR presented in (a). The data was recorded simultaneously to the differential conductance map presented in Figure 3a.

The $G(z)$ retraction curve recorded during lifting the ribbon presented in the main manuscript in Figure 3 initially reaches $G(z) \sim 0.1G_0$. The conductance lowers with increasing tip sample separation, but does not follow the exponential decay characteristic of semi-conducting ribbons.

Curved ribbon due to pentagon defects on one side

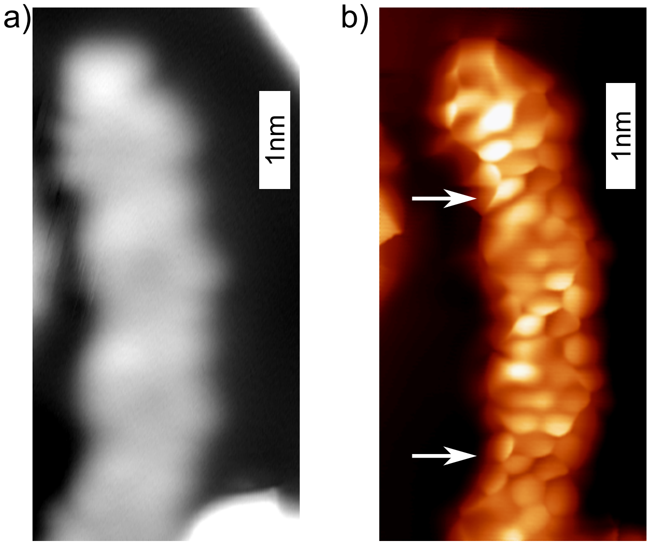

Figure 4: (a) STM topography image ($V = -300\,\mathrm{mV}$, $I = 30\,\mathrm{pA}$) of a curved ribbon. (b) Bond-resolved constant height current image ($V = 5\,\mathrm{mV}$) of the same ribbon. The 2B-units alter the contrast due to buckling of the ribbon. Pentagons are indicated by white arrows.

The ribbon presented in Figure 4 of the main manuscript exhibits the pentagon defects in an alternating fashion, creating a straight ribbon. We also observe ribbons with pentagon defects on only one side, resulting in a curvature of the ribbon. We find no clear preference for one or the other configuration. Nevertheless, curved ribbons were avoided in the transport experiments, excluding the presence of pentagon defects on only one side for the measured ribbons.

Transition from Kondo to IET-regime

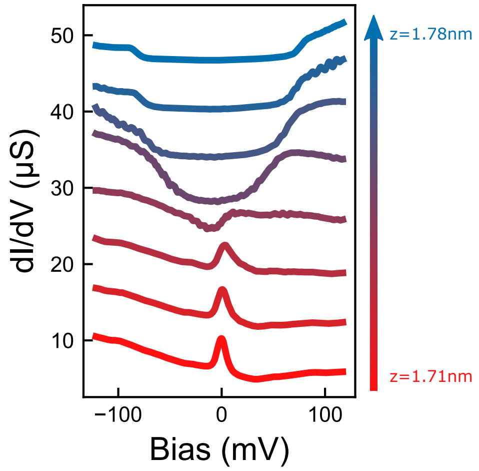

Figure 5: A Stack of differential conductance spectra taken in steps of $\Delta z = 10\,\mathrm{pm}$ shows the continuous transition from the Kondo resonance to a IET excitation. Data corresponding to Figure 4e of the main manuscript. The spectra are offset by $6.6\,\mu\mathrm{S}$ for clarity.

The ribbon presented in Figure 4 of the main manuscript undergoes a transition from Kondo regime to an IET regime upon tip retraction starting from $z \sim 1.7\,\mathrm{nm}$. The transition takes place smoothly during few tens of pm.

The Four Sites Fermi-Hubbard Model

The finite-size Fermi-Hubbard model with n sites has the following Hamiltonian:

$$\hat{H} = \sum_{j=1}^{n-1} \sum_{\sigma=\downarrow}^{\uparrow} t \left(\hat{c}^{\dagger}_{j+1,\sigma} \; \hat{c}_{j,\sigma} + \hat{c}^{\dagger}_{j,\sigma} \; \hat{c}_{j+1,\sigma} \right) + \sum_{j=1}^{n} U \; \hat{n}_{j,\uparrow} \; \hat{n}_{j,\downarrow}$$

where each $\hat{c}^{\dagger}_{j,\sigma}$ ($\hat{c}_{j,\sigma}$) is the fermionic creation (annihilation) operator of site j and spin σ, $\hat{n}_{j,\sigma} = \hat{c}^{\dagger}_{j,\sigma} \; \hat{c}_{j,\sigma}$, U is the Hubbard on-site repulsion between two electrons and t is related to the kinetic energy of an electron hopping from one site to an adjacent site. A schematic illustration of the structure is shown in the main manuscript in Figure 4g. Approximate values of the two parameters were obtained from the band structure shown in Figure 4f of the main manuscript. The energy spectrum and eigenstates of the system were obtained through exact diagonalization.

To analyze the stability of the Fermi-Hubbard model with respect to changes of U, we have considered U values ranging from 0 to $2t$. The results are presented in Figure 6. Within this energy range, no qualitative change of behaviour is observed. The energy difference between the ground state and the two excited states remains in the order of t, indicating that this is the relevant energy scale for the experimentally observed excitations. Furthermore, the energy scale of the first excited states of the model is robust against changes in the number of electrons. Excitation energies were calculated for one, two and three electrons in the model (Figure 7). We find that the excitation energies match reasonably well with the experimental data in all cases.

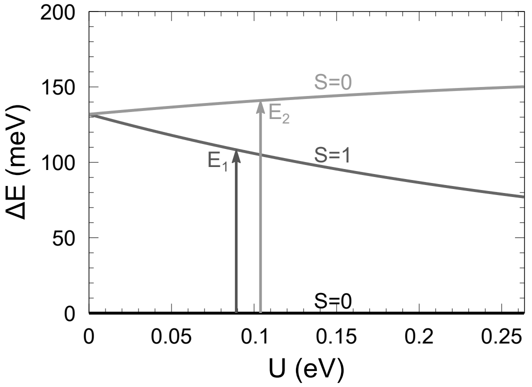

Figure 6: Excitation energy ΔE obtained from the energy spectrum of the Fermi-Hubbard model as a function of U in the two electron model with $t = 133.9\,\mathrm{meV}$.

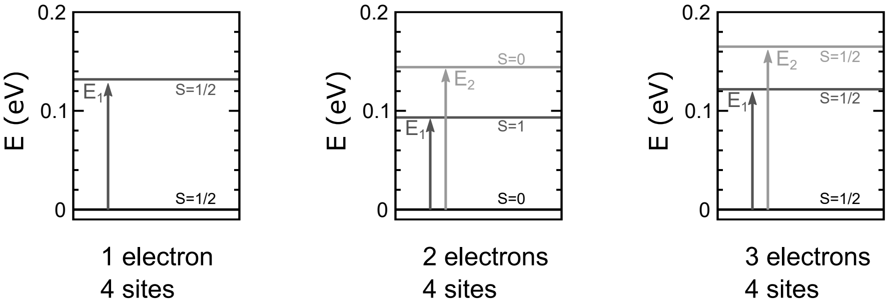

Figure 7: Excitation spectrum obtained from the four-site model Hamiltonian with one, two and three electrons (left to right) for $U = 155.9\,\mathrm{meV}$ and $t = 131.9\,\mathrm{meV}$. For an odd number of electrons, there are only doublet-doublet excitations in this energy range. For an even number of electrons, there are both singlet-triplet and singlet-singlet excitations. Note that the excitation energy increases when going from two to three electrons.

DFT Calculations

Magnetic States in Finite Ribbons

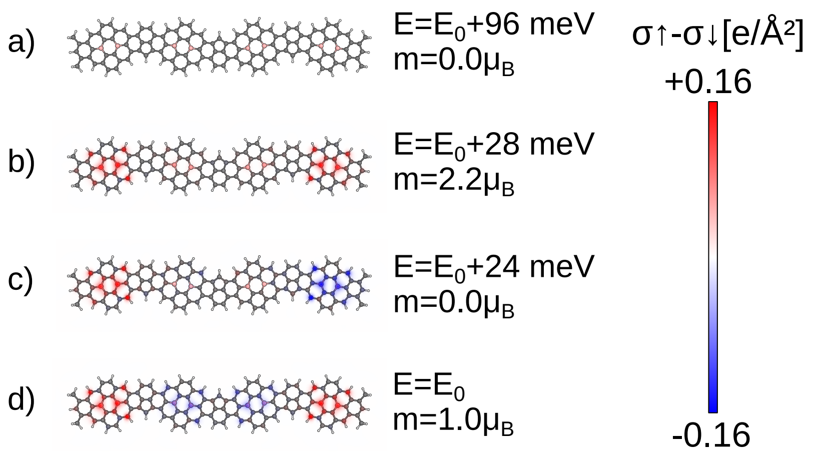

Figure 8: Spin polarization density for the finite 2B-575*-aGNR converged for different spin configurations. (a) Non-spin-polarized solution. (b) The spin in all 2B-units aligns. There is a stronger spin-polarization present at the termini. (c) The spin in the termini anti-aligns, while there is hardly any spin-polarization localized around the two inner 2B-units. (d) Ground state of the system. The spins localized in the termini align with each other and anti-align with the spin around the inner 2B-units. The spin-polarization on the two inner 2B-units does not fully compensate the magnetic moments localized at the termini.

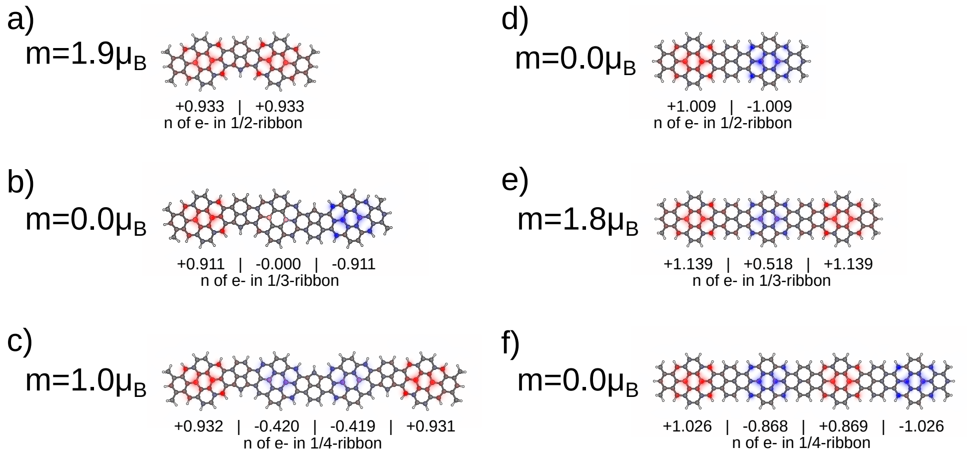

Figure 9: Spin polarization densities of the energetic ground state of each finite 2B-575*-aGNR and 2B-575-aGNR. (a),(b),(c) for finite 2B-575*-aGNR of 2, 3 and 4 2B-units, respectively. (d),(e),(f) for finite 2B-575-aGNR of 2, 3 and 4 2B-units, respectively.

Magnetic Ground State of the Periodic 2B-575*-aGNR

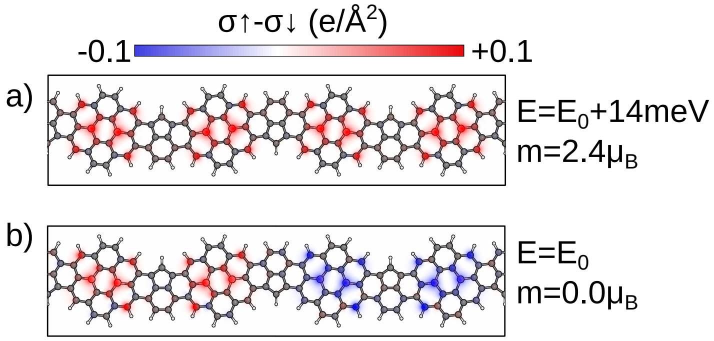

Figure 10: Spin polarization density for the periodic 2B-575*-aGNR using a doubled supercell. (a) Aligned solution. (b) Anti-aligned solution. The anti-aligned groundstate is 14 meV lower in energy.

Continuous Boron Substitution and Boron Bands Origin

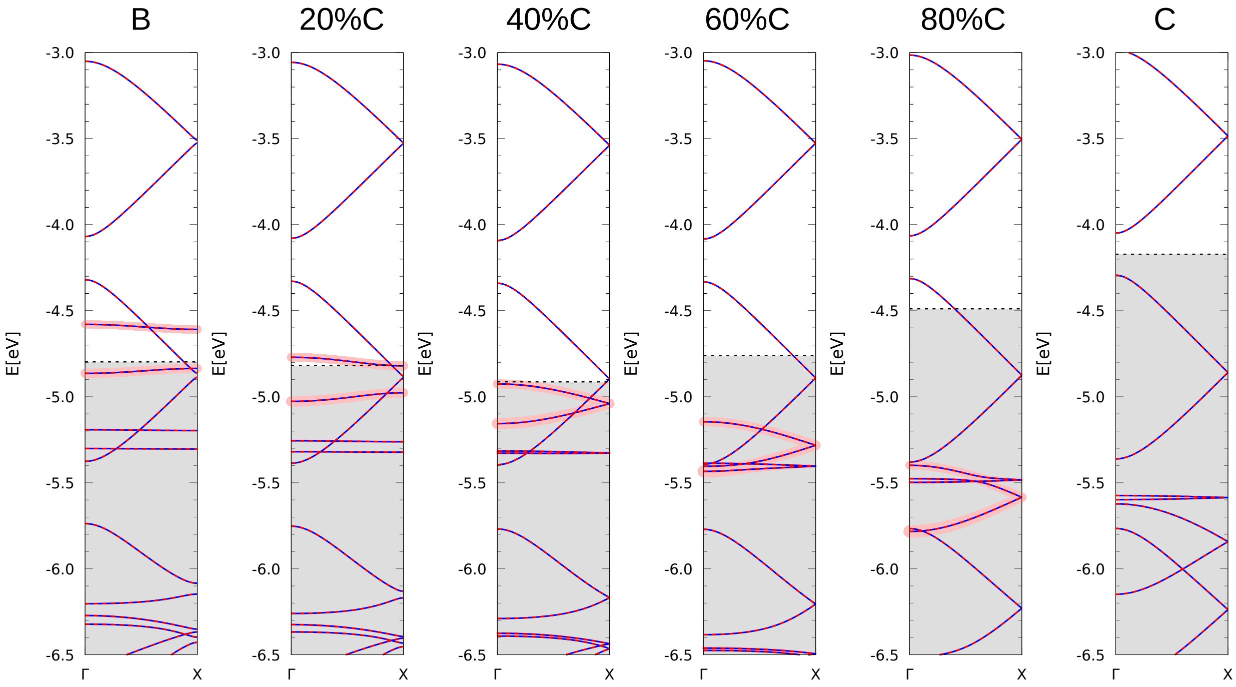

Figure 11: Electronic band structures of periodic 2B-575-aGNR as obtained from virtual crystal calculations with synthetic atoms (created as a mixture of Boron and Carbon) at the substitutional doping sites. Boron character of the bands is indicated by a pink shadow.

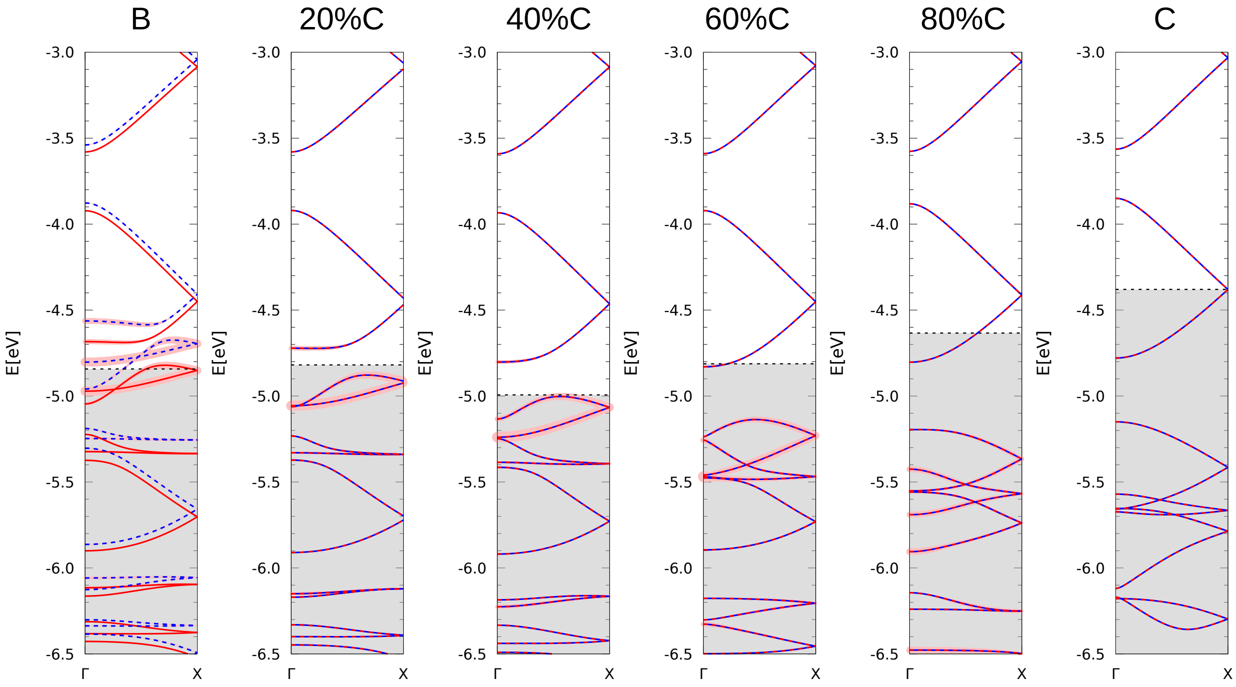

Figure 12: Electronic band structures of periodic 2B-575*-aGNR as obtained from virtual crystal calculations with synthetic atoms (created as a mixture of Boron and Carbon) at the substitutional doping sites. Boron character of the bands is indicated by a pink shadow.

Influence of the Ribbon Width Modulation

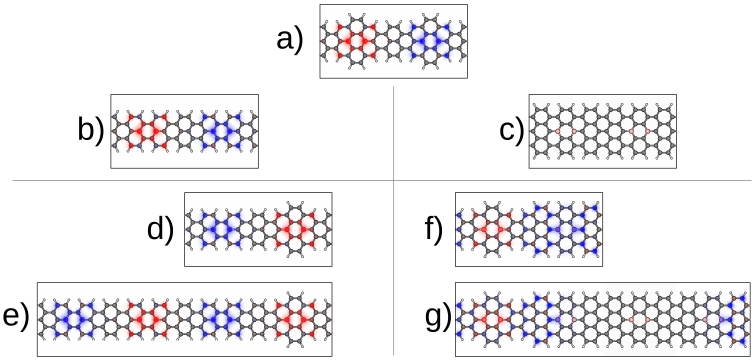

Figure 13: Spin polarization densities corresponding to different periodic systems with the same distance between substitutional doping sites. (a) 2B-575-aGNR, (b) 2B-5-aGNR and (c) 2B-7-aGNR. It is worth pointing out that 2B-5-aGNR exhibits a local magnetization very similar to the one featured in 2B-575-aGNR whereas for the 2B-7-aGNR there is no local magnetization. Panels (d),(e),(f) and (g) show mixed systems, the combination of 2B-575-aGNR with the two other GNRs. (d) presents a system combining each one 2B-unit of a 2B-5-aGNR and a 2B-575-aGNR. It presents a magnetization similar to both of their parent systems. (e) presents a system combining three 2B-units of a 2B-5-aGNR with one of a 2B-575-aGNR. (f) presents a system combining one 2B-unit of a 2B-575-aGNR followed by one of a 2B-7-aGNR. The carbon atoms located in the 7-aGNR-segments contribute to the magnetization. (g) presents a system combining one 2B-unit of a 2B-575-aGNR followed by three of a 2B-7-aGNR. Local magnetization goes to zero for the 2B-7-aGNR unit located the furthest away from the 2B-575-aGNR.

Complete Set of Bands of the 2B-575-aGNR Near the Fermi Level

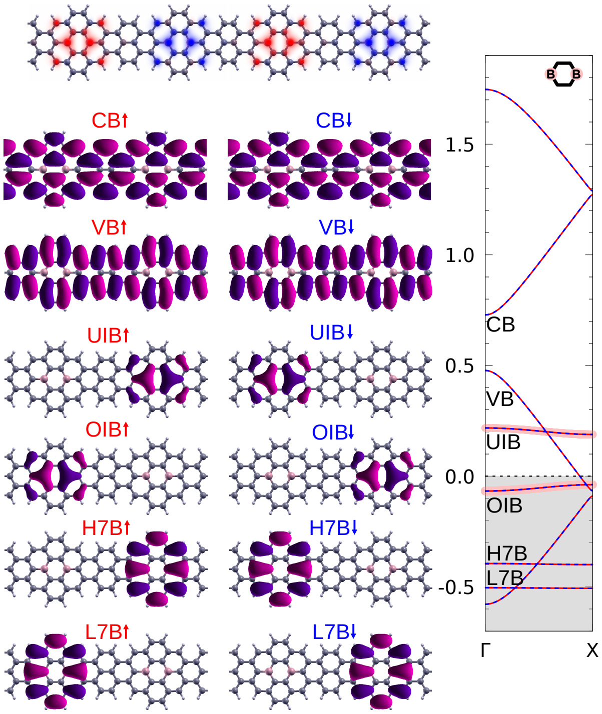

Figure 14: DFT calculated wavefunctions at Γ for the bands near the Fermi level of the 2B-575-aGNR. The wavefunctions for the most dispersive bands (CB and VB) are identical for spin up and down cases. The weakly dispersive bands (UIB, OIB, H7B and L7B) exhibit different wavefunctions depending on the spin.

Bibliography

1. Bieller, S.; Zhang, F.; Bolte, M.; Bats, J. W.; Lerner, H.-W.; Wagner, M. Bitopic Bis-and Tris (1-Pyrazolyl) Borate Ligands: Syntheses and Structural Characterization. *Organometallics* **2004**, *23*, 2107–2113.
